# Normative Values for Intertrial Variability of Motor Responses to Nerve Root and Transcranial Stimulation: A Condition for Follow-Up Studies in Individual Subjects

**DOI:** 10.1371/journal.pone.0155268

**Published:** 2016-05-16

**Authors:** Walter Troni, Federica Melillo, Antonio Bertolotto, Simona Malucchi, Marco Capobianco, Francesca Sperli, Alessia Di Sapio

**Affiliations:** 1 Service of Neurology and Clinical Neurophysiology, Clinica Fornaca di Sessant, Turin, Italy; 2 2nd Department of Neurology, Multiple Sclerosis Regional Centre, San Luigi Gonzaga Hospital, Orbassano, Italy; University of Szeged, HUNGARY

## Abstract

**Objective:**

Intertrial variability (ITV) of motor responses to peripheral (CMAP) and transcranial (MEP) stimulation prevents their use in follow-up studies. Our purpose was to develop strategies to reduce and measure CMAP and MEP ITV to guide long-term monitoring of conduction slowing and conduction failure of peripheral and central motor pathway in the individual patient.

**Methods:**

Maximal compound muscle action potentials to High Voltage Electrical Stimulation (HVES) of lumbo-sacral nerve roots (r-CMAP) and activated, averaged motor evoked potentials (MEPs) to Transcranial Magnetic Stimulation (TMS) using double cone coil were recorded from 10 proximal and distal muscle districts of lower limbs. The procedure was repeated twice, 1–2 days apart, in 30 subjects, including healthy volunteers and clinically stable multiple sclerosis patients, using constant stimulating and recording sites and adopting a standardized procedure of voluntary activation. ITV for latency and area indexes and for the ratio between MEP and r-CMAP areas (a-Ratio) was expressed as Relative Intertrial Variation (RIV, 5^th^-95^th^ percentile). As an inverse correlation between the size of area and ITV was found, raw ITV values were normalized as a function of area to make them comparable with one another.

**Results:**

All RIV values for latencies were significantly below the optimum threshold of ± 10%, with the exception of r-CMAP latencies recorded from Vastus Lateralis muscle. RIVs for a-Ratio, the most important index of central conduction failure, ranged from a maximum of -25.3% to +32.2% (Vastus Medialis) to a minimum of -15.0% to + 17.4% (Flexor Hallucis Brevis).

**Conclusions:**

The described procedure represents an effort to lower as much as possible variability of motor responses in serial recording; the reported ITV normative values are the necessary premise to detect significant changes of motor conduction slowing and failure in the individual patient in follow-up studies.

## Introduction

Electrophysiological testing is widely used to assess peripheral and central motor pathways and, in repeated determinations, it could assist clinical monitoring, providing objective and quantitative data. In follow-up studies in patient populations conventional statistical analysis, such as paired *t-*test, can demonstrate a significant variation in time of any neurophysiological index. On the contrary, the same goal in the individual patient requires a precise knowledge of normal intertrial variability (ITV) of the same index repeatedly assessed in subsequent recording sessions. This need is particularly felt in current clinical management of patients with chronic diseases, like Multiple Sclerosis (MS), in whom both clinical examination and neuroimaging gradually lose sensitivity over time [1,2].

In the assessment of motor pathways, conduction failure, i.e. the variable association of conduction block, axonal damage and neuronal death, reflects the degree of motor impairment more reliably than conduction slowing and is expressed by the area of motor responses [3], both in central as well as in peripheral diseases. Amplitudes and areas of motor responses, in particular of motor evoked potentials (MEPs), fluctuate much more than latencies [3–10]. As a consequence, the actual methodological challenge is to develop techniques to improve intertrial stability of motor areas. Magistris et al. [3] suggested that variability of MEP area is mainly due to phase cancellation phenomena of the action potentials caused by a variable degree of desynchronization along the cortico-spinal pathway and at the spinal cell level. These authors proposed the “triple stimulation technique” (TST), which allows MEP resynchronization and provides a reliable comparison between MEP and CMAP areas recorded from the same site.

So far few efforts have been devoted to quantify ITV of motor *areas* to peripheral and central stimulation in subsequent recording sessions. Only few studies dealt with ITV of MEP [11–14] or CMAP *amplitude* [15,16]. However, amplitude is of very little use in clinical application in evaluating temporally dispersed motor responses.

We have demonstrated that the combined use of high voltage electrical stimulation (HVES) of lumbo-sacral roots, using the dorso-ventral montage [17], and Transcranial Magnetic Stimulation (TMS), using the double cone coil, allows a simultaneous and bilateral recording of several root-CMAPs (r-CMAP) and MEPs from the same recording sites of lower limbs [18,19]. This diagnostic procedure allows extensive mapping of central and peripheral motor function in proximal and distal districts of the lower limbs using a *single* cortical and a *single* vertebral stimulation site. In particular, we have shown that MEP area, although partly biased by variable degrees of phase cancellation, is not a so untamable neurophysiological parameter: intrasession variability of MEP area can be significantly reduced by averaging few MEPs activated through an objective and controlled approach [19].

In this study, to exploit the technique also as a monitoring tool, we applied the described method twice, 1–2 days apart, in normal subjects and in clinically stable MS patients to obtain normative values for ITV of latencies and areas of motor responses to root and transcranial stimulation. To reduce ITV, we developed strategies to detect and control the main methodological and technical factors contributing to variability.

Ambient temperature is the most important physical factor influencing neuromuscular electrophysiology [20]. Also circadian changes of body temperature result in small but significant variations of peripheral conduction velocity [21] and, necessarily, also of central conduction velocity. This implies the need for a careful control of skin temperature and suggests performing retesting approximately at the same time.

Although occasionally reported as an expected cause of variability [22,23], the effect of displacement of the recording site on ITV of CMAP or MEP shape and area has never been quantified. In this study we faced this important factor of variability and suggest a simple approach to maintain constant all recording sites without time limits.

A novel feature of our results was that raw ITV values for areas include a significant component inversely related to the area size. We removed this bias, which prevents a direct comparison of ITVs of responses of different size, through a normalization procedure.

## Materials and Methods

### Preliminary study: effect of shift of the recording site on ITV of CMAP latency and area

**Fig 1:**
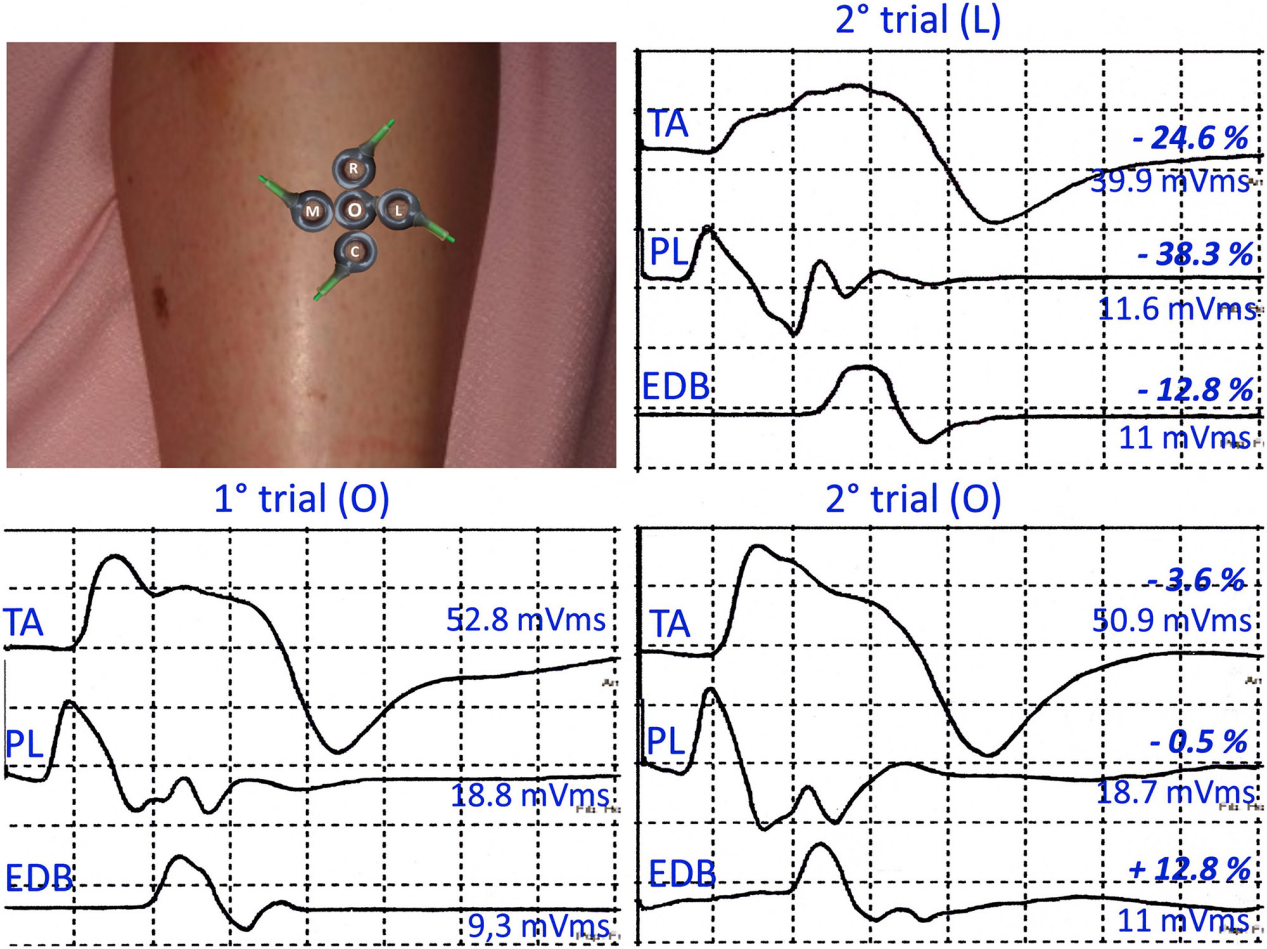
Assessment of the effects of changing the recording site on ITV of CMAP area and latency. Single maximal CMAPs recorded from Tibialis Anterior (TA), Peroneus Longus (PL) and Extensor Digitorum Brevis (EDB). Recording was repeated twice the following day a) using the same recording site (O) or b) randomly moving the cathode 1 cm medial (M), lateral (L), rostral (R) or caudal (C) to O, as shown in the figure for TA. Note the significant ITV reduction of CMAP area using the same recording site and the precise replication of the morphological features of individual CMAPs.

In 10 normal volunteers, ranging in age between 28 and 67 years, we studied the effects of small shifts of the recording cathode on CMAP latency and area according to the following procedure (Fig 1): during the first recording session maximal CMAPs to supramaximal stimulation of the peroneal nerve at the knee were simultaneously recorded from Tibialis Anterior (TA), Peroneus Longus (PL) and Extensor Digitorum Brevis (EDB) muscles after locating the recording sites giving the optimal responses (CMAPs of maximal amplitude, regular shape and sharp negative onset). The following day, at approximately the same time and after control of the skin temperature, the procedure was repeated twice: a) placing the cathode on the same recording site and b) after moving it to a new position, randomly chosen, 1 cm rostral, caudal, medial or lateral to the original site, as shown in Fig 1 for TA.

Latencies and areas of all rectified CMAPs were measured after baseline correction and ITV values were calculated for each couple of responses, V1 and V2, recorded with identical or changed recording sites, according to the formula 100(V2-V1)/0.5(V1+V2) (see section “statistical analysis”).

**Fig 2:**
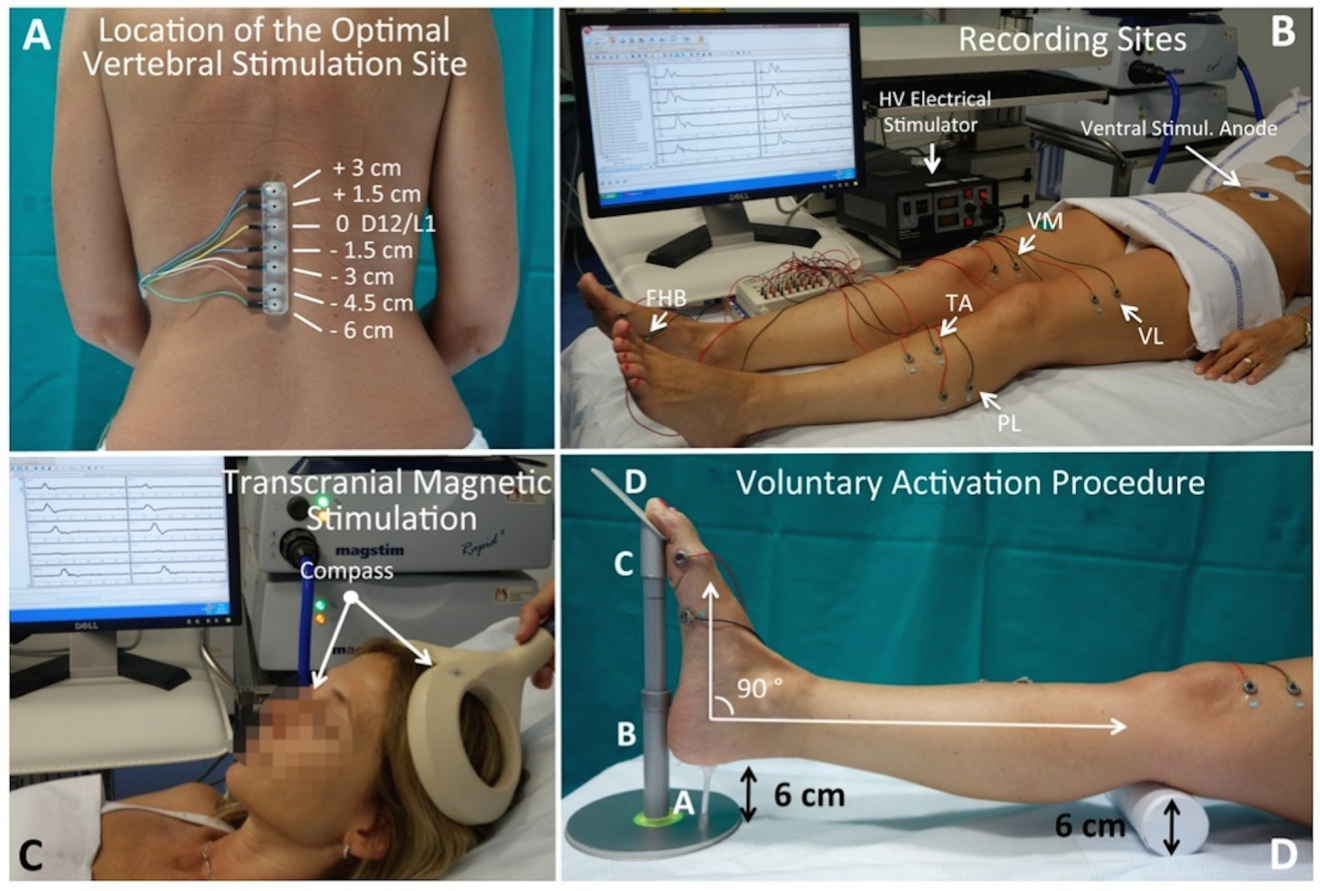
Neurophysiological mapping of central and peripheral motor function in lower limbs. CMAPs to root stimulation (r-CMAPs) using the dorsoventral montage (Fig 2A) were simultaneously recorded from proximal and distal muscles of both lower limbs (VM, Vastus Medialis; VL, Vastus Lateralis; TA, Tibialis Anterior; PL, Peroneus Longus; FHB, Flexor Hallucis Brevis) (Fig 2B). The optimal vertebral stimulation site was located by testing several sites over the dorso-lumbar junction of the spinal cord by means of a multielectrode (Fig 2A). MEPs to TMS using the double cone coil were recorded from the same sites. The correct position of the coil was reproduced by measuring the distance between nasion and the anterior edge of the coil (Fig 2C). Simultaneous and controlled voluntary activation of all muscle districts was obtained by asking the subject to perform a sequence of 3 movements of fixed amplitude and to maintain the reached position with the aid of a simple purpose-made device (Fig 2D: further explanation in the text).

All responses, in this and in all the following procedures, were recorded using sintered Ag/AgCl Multitrode^®^ surface electrodes (Brain Product GmbH, Munich, Germany; Fig 2). These non-polarizable ring-shaped electrodes can be easily attached to bare skin by double-side, adhesive rings. The large central opening (6 mm diameter) makes easy skin treatment and gel filling to effectively reduce impedance below 5 kOhm.

### Subjects

The study was conducted on 30 subjects in accordance with the Declaration of Helsinki: all subjects gave written consent before participating and the study was approved by the local ethical committee (Comitato Bioetico dell’Ospedale San Luigi Gonzaga, Orbassano, Torino).

The casuistry was equally composed by normal subjects (9 females and 6 males; mean age 43 years, range 24–68) and MS patients (8 females and 7 males; mean age 39 years, range: 26–60). Four patients had a clinically isolated syndrome, 10 had a relapsing remitting MS and 1 a primary progressive MS; all patients were clinically stable for at least 6 months. The mean EDSS was 0.9 (range 0–2.5); most patients showed a mild pyramidal impairment: the mean Pyramidal Functional Score was 0.6 (range 0–2).

### Root CMAP and MEP recording

#### High voltage electrical stimulation (HVES) of lumbo-sacral nerve roots

Maximal CMAPs, elicited by HVES of the lumbo-sacral roots (r-CMAP, Fig 2A) using the dorso-ventral montage [17,18], were simultaneously recorded by means of pair of Multitrode^®^ electrodes (placed 4 cm apart in belly-tendon montage) from 5 muscle districts of both sides in a proximal-distal arrangement: Vastus Medialis (VM), Vastus Lateralis (VL), Tibialis Anterior (TA), Peroneus Longus (PL) and Flexor Hallucis Brevis (FHB) (Fig 2B). The optimal recording sites were located according to the procedure previously described [17]. The correct vertebral stimulation site was identified during the first session by using a multiple electrode array placed over the dorso-lumbar tract of the spinal cord (Fig 2A). It was defined as the most rostral site where a stimulus just above threshold elicited submaximal responses in proximal and distal recording sites simultaneously [18]. HVES was performed by using a high voltage electrical stimulator Digitimer D 185-Mark IIa (Digitimer Ltd., UK), with a maximum output up to 1000 V, which produces a rectangular (50 μs) pulse shape with extremely rapid rise and fall times. The current intensity was progressively increased to achieve r-CMAPs of maximal amplitude in all recording sites as proved by complete saturation of responses. Maximal r-CMAPs were reached with stimulus intensities not exceeding 600 V (1100 mA). Latencies of r-CMAPs were used as peripheral conduction time (PCT).

#### TMS and voluntary activation

MEPs were elicited using the double cone coil (110 mm diameter), very effective to stimulate bilaterally the deep cortical regions projecting to the lower limbs [24,25]; it was placed on a midline position, 2 cm behind the vertex (Fig 2D). Magnetic stimuli were delivered through a Magstim^®^ Rapid device (The Magstim Company Ltd, Whitland, UK, 0.5–1.4 Tesla). First, we defined the motor threshold as the stimulus intensity giving a MEP with amplitude ranging from 50 to 200 μV in at least 5 of the 10 recording sites; then, we delivered stimuli 150% above the threshold. In each subject 2–3 basal MEPs and 5 activated MEPs were obtained.

Voluntary activation was performed according to the following procedure. We tried to obtain a reproducible degree of voluntary muscle activation simultaneously in all tested muscles by asking the subject to perform a predetermined pattern of movements of defined amplitude [19]. As shown in Fig 2D, a 6 cm diameter round, rigid cylinder was placed under the popliteal fossa of both legs of the supine subject. He/Her was asked to perform a rapid sequence of 3 movements simultaneously with both legs to reach a predetermined position: (1) thigh extension to lift the heels 6 cm over the bed to activate VM and VL; (2) dorsal flexion of the foot to form a 90° angle between sole and tibial axis to activate TA and PL; (3) plantar flexion of the hallux of 25°/30° to activate FHB. To control the correctness of each movement and the maintenance of the reached position, two identical devices were placed medially to each ankle with the subject in relaxed position (Fig 2D): a light contact of the heel skin with point A, of the sole with points B and C, and of the hallux with point D assured the correct degree of thigh extension, foot dorsiflexion and hallux flexion, respectively. Support A, made of flexible rubber, was designed to allow a simple skin contact, and not to support the weight of the leg. A short training was sufficient to perform the motor task properly. After the examiner verified the correct position, TMS was delivered about 1–2 s after a verbal warning. After TMS the subject was asked to return to the relaxed position and the same procedure was repeated at intervals of approximately 30 sec.

To avoid any possible conditioning effect of the previous root stimulation on excitability of cortical motor area, we interposed a long time interval between HVES and TMS (10 minutes), exceeding far away any known duration of conditioning effect exerted by single stimuli [26]. To further verify this assumption, in 5 normal volunteers (including authors) activated MEPs (see below) from all recording sites were obtained before and 10 minutes after HVES and their area values were compared.

#### Electrophysiological indexes: recording and analysis

A 16 channel bipolar amplifier (BrainAmp ExG, Brain Products GmbH, Germany) was used for r-CMAPs and MEPs recording. Signals were filtered (10–1000 Hz) and amplified. Data were examined on-line and saved for subsequent analysis. Latencies (ms) and areas (mVms) of all motor responses were calculated off-line after baseline correction and rectification. The average of the 5 rectified and activated MEPs was used for analysis.

The following electrophysiological indexes were assessed in both sides for each of the 5 recording sites:

1. Latency of r-CMAP, as a measure of the PCT
2. Latency of averaged MEP
3. Central Motor Conduction Time (CMCT), resulting by subtracting PCT to MEP latency
4. Area of r-CMAP
5. Area of averaged MEP
6. a-Ratio, i.e. the ratio between MEP and r-CAMP areas, which expresses the fraction of r-CMAP recruited by TMS

### Procedure of short term retesting

**Fig 3:**
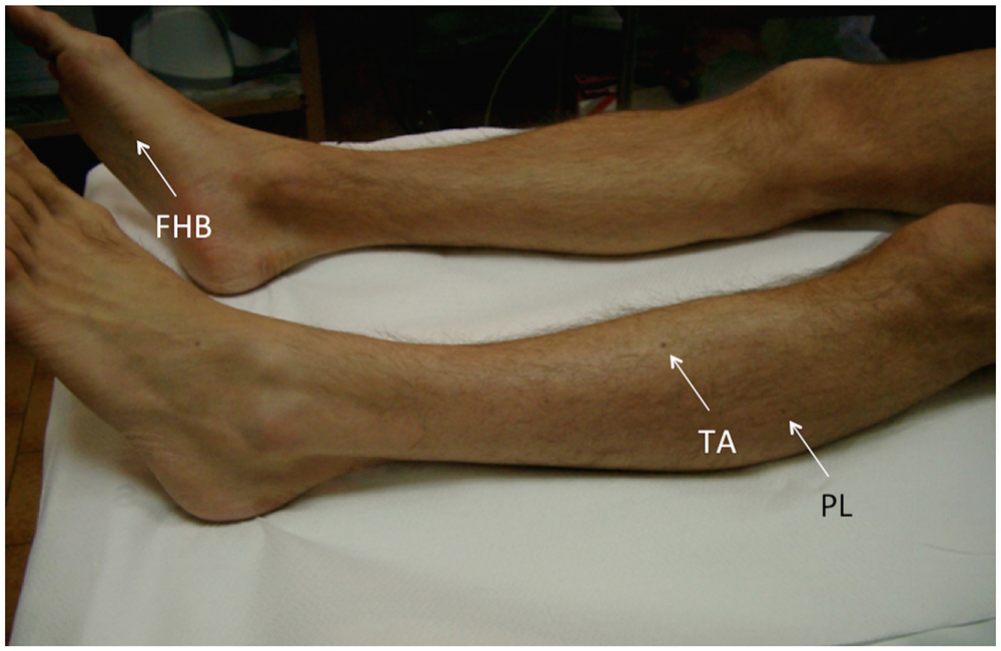
Marking of recording and stimulating sites. In selected patients for whom a long-term neurophysiological follow-up was planned, all recording sites and the stimulation site on the vertebral column were tagged with a small, indelible skin mark.

All experiments were carried out at a room temperature set at 22°C. Skin temperature at two sites, proximal (anterior face of thigh) and distal (sole of feet), was recorded. At the end of the first recording session, a small round mark centered on the circular opening of the electrode was made with a dermographic pen on all recording sites and on the stimulating site over the vertebral column. Selected patients, for whom a long-term follow-up was planned, received an indelible skin mark, i.e. a small round tattoo as commonly performed in radiotherapic protocols [27] (Fig 3), to allow a long-term replication of the same recording and stimulation sites. The position of the double cone coil was reproduced by measuring with a compass the distance between the nasion and the anterior edge of the coil (Fig 2C).

The same recording procedure was repeated 1 or 2 day apart, approximately at the same time, after controlling skin temperature and after careful replication of all recording and stimulation sites. The same stimulating parameters were used in the two sessions for HVES and TMS.

### Statistical analysis

Individual ITV values were calculated according the following formula, which expresses the ratio percentage of the difference between two subsequent determinations (V1 and V2) and their mean value: 100 (V2-V1) / 0.5 (V1 + V2), where V2 and V1 are the second and first determination respectively [28].

Paired *t*-test was used to compare MEP areas recorded from TA with and without cathode displacement. The same test was applied to compare MEP areas obtained before and after HVES and to evaluate reproducibility of facilitation in repeated determinations (expressed as activated MEP area/basal MEP area). Differences among ITVs of neurophysiological indexes of MS patients and normal volunteers were evaluated using the Student's *t*-test. Differences with a *p* value less than 0.05 were selected as significant in all the comparisons.

Correlations between area and latency values of r-CMAP and MEP with the respective absolute ITV values were studied with Spearman's test.

The component of variability inversely related to area (see results) was removed according a normalization procedure described in S1 Appendix (see discussion for details).

ITV values of latencies and normalized ITV values of areas were used to calculate the Intraclass Correlation Coefficient (ICC) to quantify the reliability and reproducibility of intertrial assessment [28,29]; values greater than 0.7 were considered as reliable measurements.

Since we observed a non gaussian distribution of absolute ITV values of latency and area of MEP and r-CMAP, the normative ITV values were expressed as Relative Intertrial Variation (RIV), which indicates the range from the 5th percentile to the 95th percentile.

Data were acquired and analyzed by SPSS version 21.0 (SPSS Inc., Chicago, IL).

## Results

### Preliminary study: effect of changing the recording site on ITV of CMAPs latency and area

ITV values for latency and area of TA, PL and EDB CMAPs as a function of the position of the recording electrodes are shown in Table 1. For all muscles, ITV of CMAP areas recorded from the same site was significantly lower, about half, than that observed after random cathode displacement (*p*< 0.001; paired *t-*test). Moreover, as exemplified in Fig 1, the use of a stable recording site resulted in a faithful replication of the morphological features of individual CMAPs. The same was true for ITV of CMAP latencies.

**Table 1:** Intertrial variability of CMAP latency and area as a function of the recording site.

	A	B	C
	**Latency**		
	**mean ± SD (ms)**	**ITV % (mean ± SD)**	**ITV % (mean ± SD)**
**TA**	4.4 ± 0.4	0.2 ± 4.8	-0.7 ± 9.0
**PL**	3.0 ± 0.3	0.2 ± 4.5	-0.3 ± 8.8
**EDB**	10.2 ± 0.1	-0.3 ± 2.8	0.7 ± 4.8
	**Area**		
	**mean ± SD (mVms)**	**ITV % (mean ± SD)**	**ITV % (mean ± SD)**
**TA**	42.3 ± 20.2	1.1 ± 13.1	2.1 ± 25.3
**PL**	27.5 ± 12.2	-1.5 ± 15.2	1.8 ± 29.7
**EDB**	26.5 ± 12.7	0.9 ± 16.1	-1.5 ± 30.5

Intertrial variability (ITV%) of latency and area of Tibialis Anterior (TA), Peroneus Longus (PL) and Extensor Digitorum Brevis (EDB) CMAPs, elicited by stimulation of the peroneal nerve at knee. A: basal determination; B: determination made using the same recording site. C: determination made after changing the recording sites.

### Intertrial variability of r-CMAP and MEP latencies and areas

The first recording session was more time consuming (50–70 min). The second session took no more than 20–30 minutes, being already defined all recording and stimulating sites as well as the proper stimulus intensities. No significant differences were observed between healthy controls and MS patients for mean area values not only, as expected, of r-CMAPs but also of MEPs, confirming the slight motor impairment of patients of our casuistry; the same was true for ITVs of CMCT, r-CMAP area, MEP area and a-RATIO *(p* = 0.07, 0.48, 0.06, 0.96 respectively). Consequently, all data have been pooled and analyzed together.

Paired *t-*test did not show significant differences between MEP area recorded before and after HVES (*r* = 0.95; *p* = 0.08), ruling out any possible conditioning effects of the preceding HVES on subsequent TMS.

**Fig 4:**
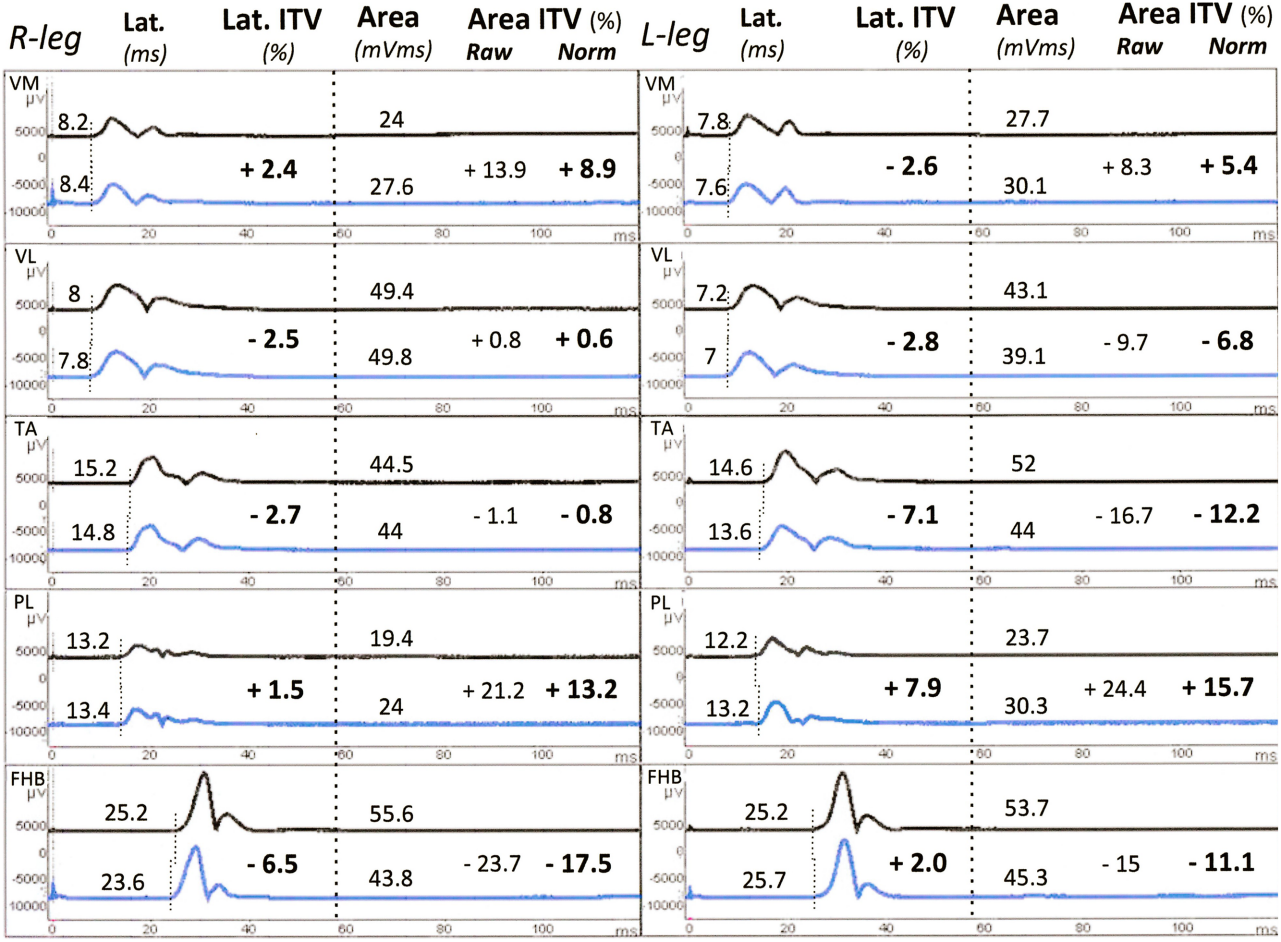
Maximal CMAPs to HVES. Subject n.25 (M.L., f, 32 ys). Rectified, single r-CMAPs bilaterally recorded from all sites during the first (upper trace) and second (lower trace) recording session. Latencies, Latency ITVs, Areas, raw and normalized Area ITVs are listed for each couple of responses. All normalization procedures were performed using custom made Excel^®^ sheets. Note the stability of latencies and the faithful replication of individual morphological features of all responses. VM, vastus medialis; VL, vastus lateralis; TA, tibialis anterior; PL, peroneus longus; FHB, flexor hallucis brevis.

**Fig 5:**
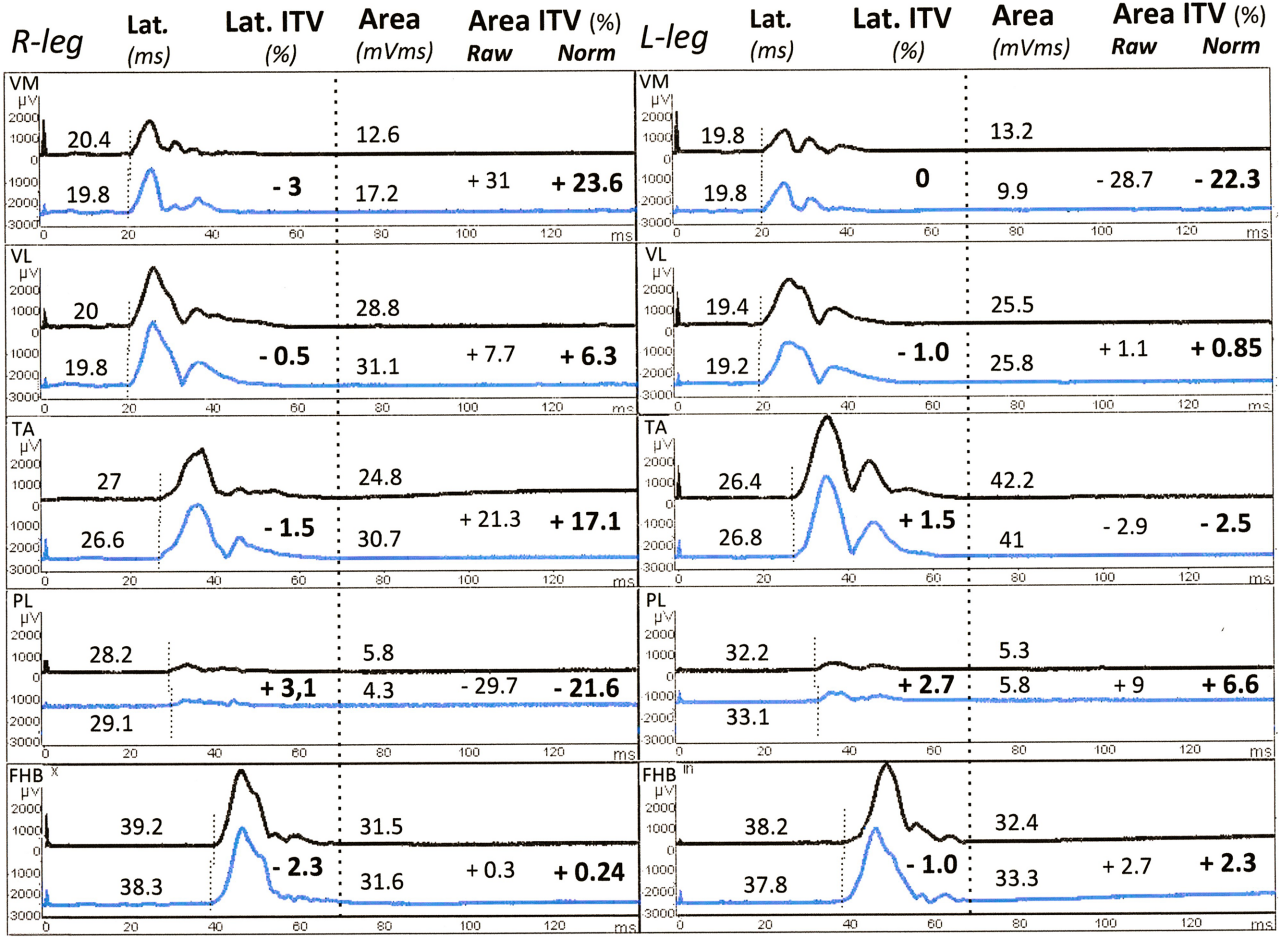
Averaged MEPs to TMS. Subject n.25 (M.L., f, 32 ys). Averaged and rectified activated MEPs bilaterally recorded from all sites during the first (upper trace) and second (lower trace) recording session. Latencies, Latency ITVs, Areas, raw and normalized Area ITVs are listed for each couple of responses. As for CMAPs, note the stability of latencies and the precise replication of the individual shape of all responses. Further details in legend of Fig 4. VM, vastus medialis; VL, vastus lateralis; TA, tibialis anterior; PL, peroneus longus; FHB, flexor hallucis brevis.

Examples of r-CMAPs and MEPs recorded in the 2 sessions in a representative normal subject are shown respectively in Figs 4 and 5. All individual values of r-CMAP and MEP latencies and areas are listed in S2 Appendix (- database.xlsx).

#### Reproducibility of the procedure of voluntary muscle activation

**Fig 6:**
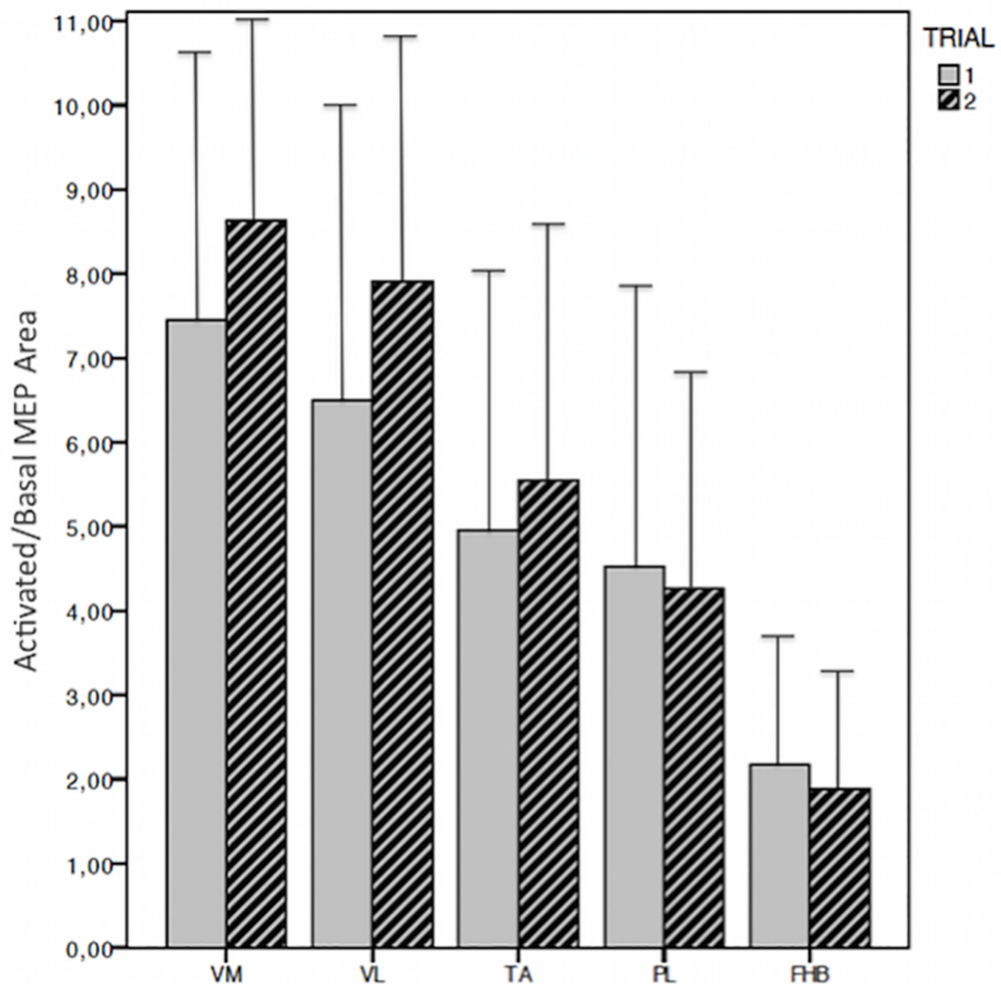
Reproducibility of voluntary MEP activation in subsequent trials. The degree of voluntary activation, expressed as the ratio between activated and basal MEP area, was similar in the two recording sessions in all tested muscles; in the different recording sites, the degree of facilitation showed a proximal-distal gradient, ranging from a maximum value of 7.3 (1st trial) and 8.6 (2nd trial) for VM to a minimum value of 2.2 (1st trial) and 1.9 (2nd trial) for FHB. VM, vastus medialis; VL, vastus lateralis; TA, tibialis anterior; PL, peroneus longus; FHB, flexor hallucis brevis.

The degree of voluntary facilitation, expressed by the ratio between averaged activated and basal MEP area, did not show any significant difference between the two trials for all recording sites and showed the same proximal-distal gradient (Fig 6), as previously reported [19].

#### ITV and RIV for r-CMAP and MEP latencies and for CMCT

In Table 2 are reported mean values ± SD for r-CMAP and MEP latencies and for CMCT (right and left values pooled together) recorded in the first and second session from all muscle districts with respective RIV values. All RIVs for r-CMAP and MEP latencies, with the exception of VL r-CMAP, were below ± 10%. CMCT RIVs ranged from -14.9% to +16%.

**Table 2:** Root-CMAP latencies, MEP latencies and CMCTs.

**Recording Site**	**r-CMAP 1 (ms) mean ± SD**	**r-CMAP 2 (ms) mean ± SD**	**RIV**
**VM**	9 ± 0.8	9 ± 0.9	-9.6 / +9.1
**VL**	8.7 ± 1.4	8.7 ± 1.5	-9.7 / +13.6
**TA**	16 ± 1.4	16 ± 1.5	-6.4 / +5.2
**PL**	14.6 ± 1.3	14.5 ± 1.4	-6.7 / +6.9
**FHB**	24.6 ± 2	24.3 ± 1.9	-6.5 / +4.1
	**MEP 1 (ms) mean ± SD**	**MEP 2 (ms) mean ± SD**	**RIV**
**VM**	21.6 ± 2.9	21.7 ± 3.0	-6.4 / +6.7
**VL**	21.9 ± 3.1	21.9 ± 3.2	-6.4 / +6.1
**TA**	29.7 ± 3.2	29.5 ± 3.2	-5.9 / +5.6
**PL**	28.1 ± 3.7	28.1 ± 3.6	-5.8 / +4.2
**FHB**	38.3 ± 3.7	37.8 ± 3.7	-7.6 / +3.5
	**CMCT 1 (ms) mean ± SD**	**CMCT 2 (ms) mean ± SD**	**RIV**
**VM**	12.6 ± 2.6	12.7 ± 2.7	-12.2 / +15.1
**VL**	13.3 ± 3.0	13.3 ± 3.1	-14.9 / +11.5
**TA**	13.8 ± 2.6	13.7 ± 2.5	-14.8 / +13.8
**PL**	13.6 ± 3.2	13.6 ± 3.4	-12.4 / +12
**FHB**	13.7 ± 2.8	13.6 ± 2.8	-13.1 / +16

Mean values ± SD for r-CMAP and MEP latencies and for CMCT from all muscle districts, recorded in the first and second session with respective normal ranges of intertrial variability expressed as relative intertrial variations (RIV).

ICC values were 0.997 and 0.993 for r-CMAP and MEP latency respectively and 0.958 for CMCT.

As expected, ITVs of r-CMAP latencies were inversely related to conduction distance (p = 0.003) reaching the minimum value recording from FHB (RIV: -6.5% to +4.1%), and maximum recording from VM and VL (RIV: -9.6% to +9.1%). A similar trend was observed for MEP latencies obtained from healthy subjects of the casuistry. On the contrary, within the same recording site no significant differences were observed between individual ITV values and the corresponding latency values.

#### Correlation between ITV and r-CMAP and MEP areas

**Fig 7:**
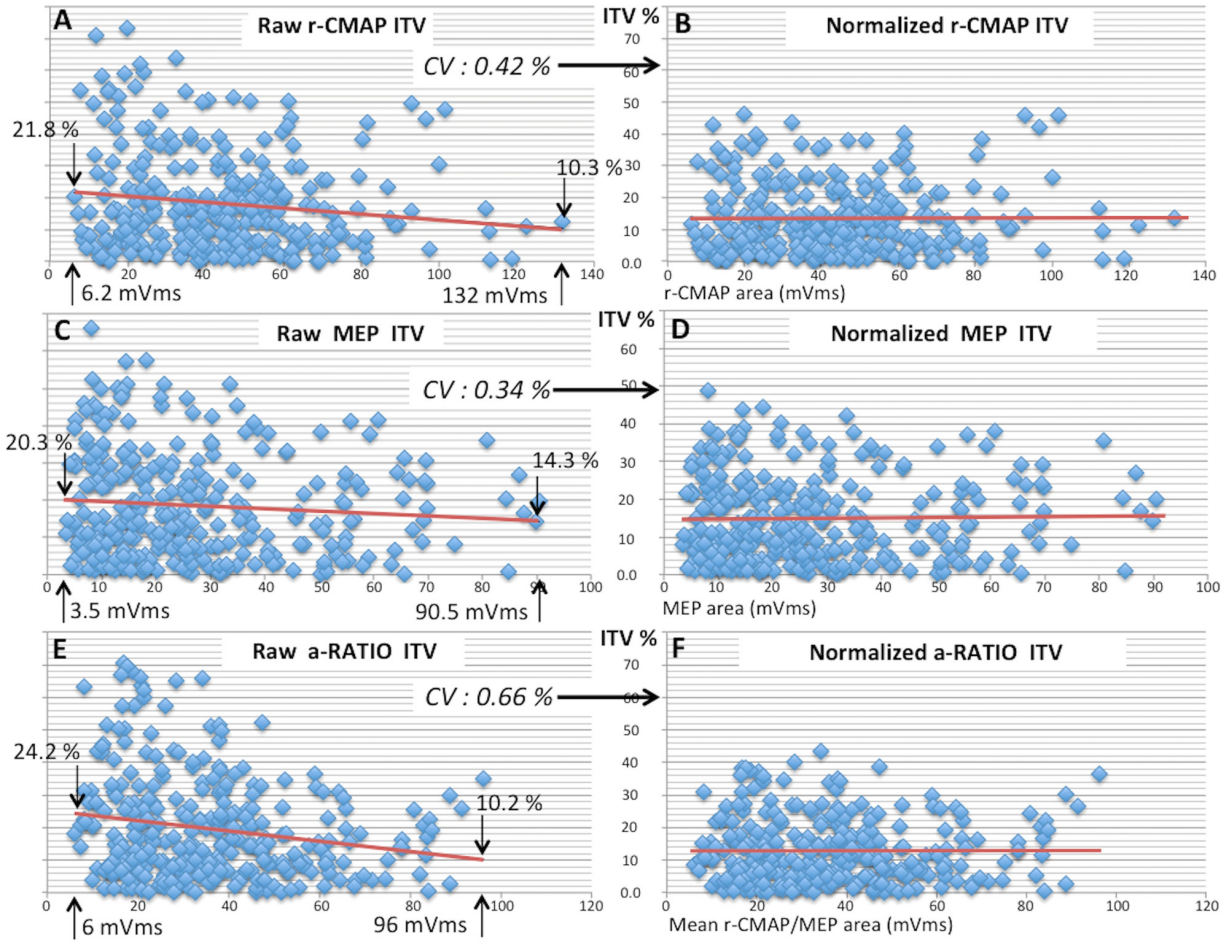
Regression lines correlating area and ITV. Regression lines correlating ITV and Area for r-CMAP (A), MEP (C) and a-Ratio (E). Note that responses of smaller area tend to have a greater ITV as expressed by the Coefficient of Variability. Normalization of raw ITV values (see S1 Appendix) resulted in a complete removal of the variability component inversely correlated to area, as shown in B, D and F for r-CMAP, MEP and a-Ratio respectively. Note that ITV correction, minimum for responses of large area, increases as a function of the decrease of area.

When the mean areas of the 2 sessions of r-CMAP and MEP (300 values) were correlated to the respective raw ITV values, an inverse correlation was found (*r* = - 0.141, *p* = 0.016 for r-CMAP and *r* = - 0.165, *p* = 0.005 for MEP), suggesting that responses of small area were exposed to a greater ITV. Since a-Ratio interrelates MEP and r-CMAP areas, its raw ITV values showed a similar inverse correlation (*r* = -0.187, *p* = 0.001). Regression line of absolute raw ITV values was even steeper for a-Ratio because this parameter was exposed to the combined ITVs, randomly concordant or opposing, of r-CMAP and MEP areas (Fig 7A, 7C and 7E). When all absolute raw ITVs (900) were correlated with respective area values a highly significant inverse relationship was observed (*r* = - 0.125, *p* = 0.000169).

This ITV component inversely related to area prevents definition of normative values common to any motor response regardless of the size of their area (see Discussion). To avoid this bias individual ITV values were corrected as a function of individual area size according to the normalization procedure described in S1 Appendix. Normalization resulted in a complete removal of the ITV component inversely correlated to area (Fig 7B, 7D and 7E).

#### ITV and RIV for r-CMAP and MEP areas and for a-ratio

Table 3 reports mean values ± SD for mean r-CMAP and MEP areas and for a-Ratio (right and left values pooled together) recorded in the first and second session from all muscle districts with respective normalized RIV values. In the first trial, VM r-CMAP (58.6 mVms) and TA r-CMAP (59.4 mVms) showed maximum area size, whereas, among MEPs, TA MEP showed the maximum area size (51.8mVms), nearly reaching that of TA r-CMAP. This was reflected by the a-Ratio (MEP/r-CMAP), which reached the highest value for TA (0.92), followed by FHB (0.77). This profile of r-CMAP and MEP areas and of a-Ratios was exactly replicated in the second trial.

**Table 3:** Root-CMAP areas, MEP areas and a-Ratios.

**Recording Site**	**r-CMAP 1 (mVms) mean ± SD**	**r-CMAP 2 (mVms) mean ± SD**	**RIV**
**VM**	58.6 ± 22.1	60.3 ± 21.1	-29.1 / +26.6
**VL**	37.2 ± 23.2	36.8 ± 19.6	-37.8 / +36.5
**TA**	59.4 ± 24.7	57.4 ± 21.8	-21.7 / +28.0
**PL**	32.5 ± 14.4	32.1 ± 13.9	-24.8 / +24.1
**FHB**	32.7 ± 18.3	31.1 ± 16.1	-23.3 / +17.5
	**MEP 1 (mVms) mean ± SD**	**MEP 2 (mVms) mean ± SD**	**RIV**
**VM**	27.6 ± 17.4	28.5 ± 17.5	-26.0 / +37.1
**VL**	16.9 ± 9.8	18.2 ± 11.0	-21.3 / +35.0
**TA**	51.8 ± 22.2	48.7 ± 20.6	-34.5 / +30.5
**PL**	15.4 ± 10.3	16.2 ± 10.3	-24.1 / +34.8
**FHB**	22.3 ± 12.8	21.3 ± 12.3	-31.7 / +24.5
	**a-RATIO 1 mean ± SD**	**a-RATIO 2 mean ± SD**	**RIV**
**VM**	0.47 ± 0.24	0.46 ± 0.20	-25.3 / +32.2
**VL**	0.54 ± 0.36	0.54 ± 0.33	-22.0 / +31.0
**TA**	0.92 ± 0.36	0.92 ± 0.42	-25.2 / +30.0
**PL**	0.51 ± 0.29	0.54 ± 0.32	-20.9 / +26.3
**FHB**	0.77 ± 0.45	0.77 ± 0.46	-15.0 / +17.4

Mean values ± SD of r-CMAP and MEP areas and of a-RATIO recorded from all muscle districts in the first and second session with respective normal ranges of intertrial variability (ITV) expressed as relative intertrial variations (RIV).

RIVs for r-CMAP and MEP areas ranged from a maximum of -37.8% to +36.5% (VL r-CMAP) to a minimum of -23.3% to +17.5% (FHB r-CMAP). RIVs for a-Ratio were slightly lower and ranged from a maximum of -25.3% to +32.2% (VM) to a minimum of -15.0% to + 17.4% (FHB), showing a trend towards a proximo-distal reduction of RIV. In particular, the FHB a-Ratio was the index with the largest inter-subject variability (SD: ± 0,45 and ± 0,46 in the 1st and 2nd trial respectively) and the least intra-subject intertrial variability.

ICC values for r-CMAP and MEP areas and for a-Ratio were 0.943, 0.968 and 0.933 respectively.

## Discussion

### Recording site and ITV

Although occasionally reported as an expected cause of variability [22,23], the effect of displacement of the recording site on area of motor responses in subsequent recordings had never been quantified. Our results confirm that change of recording site is by far the most relevant cause of variability. We found that even slight cathode displacements nearly doubled latency and area ITVs and significantly changed the shape of motor responses. In particular, the faithful replication of the morphological features of individual responses provided by a constant recording site ensures a reproducible intertrial reference point for latency reading. In short term monitoring, as it is the case in our study, the use of small pen marks is sufficient to reproduce the same recording sites. When long-term follow-up studies are planned, small tattoos, as commonly performed in radiotherapic protocols [27] are a practical and effective tool. The procedure is simple and skin marks have a negligible visual impact; the discomfort for the patient is minimal and recording can be repeated from the same sites with high precision without time limits.

### Procedure of voluntary MEP activation

The combined use of activated MEPs [30,31] and MEP averaging [14,19,32] significantly improves MEP stability. However, visual or quantitative torque control of voluntary activation [30,31] of a large number of muscles simultaneously is unfeasible. On the other hand, a voluntary activation based on the subjective feeling of the degree of contraction is unreliable when dealing with several muscles at the same time. To overcome this problem, we started from the assumption that a movement of fixed amplitude implies a constant degree of muscle contraction. The amplitude of a movement or even the sequence of several movements can be objectively controlled by an external examiner using a simple purpose-made device and can be easily replicated in repeated trials. This approach makes the degree of voluntary activation in individual muscles quite similar among subjects and almost identical in the same subject. This was confirmed for all recording sites by the similar degree of MEP facilitation in the two trials.

### Relationship between latency, area and ITVs

As expected, an inverse correlation was found between latency ITV and conduction distance, being ITV of r-CMAP and MEP from proximal districts significantly greater than that from distal ones. This is not surprising since experimental error in reading latency and in measuring the true conduction distance due to uncertain location of the real stimulating cathode is amplified or attenuated by a short or long conduction distance respectively. However, *within each recording site*, no significant correlation was found between latency and ITV. This means that the use of specific normative values as a function of the different mean conduction distances, i.e. of the different recording sites, is a correct statistical approach.

Area ITV poses a more complex methodological problem. As expected, we found that, even adopting optimal recording conditions and exploiting the best technical facilities, a residual variability component remains due to irreducible technical and biological factors. This absolute component of global variability, being expressed as a percentage variation, obviously impacts small responses much more than large ones. This finding has a relevant implication in clinical practice: individual ITV values cannot be directly compared with each other. For example, a 100% variation of a very small response of 1mVms, as it may occur in pathological cases, is unlikely to have the same clinical significance of a similar variation affecting a response of 80 mVms.

If all raw ITV values were pooled together, the resulting confidence limits to define significant or not a change in a single patient would be strongly dictated by the greater ITV of small areas, making their use unreliable for large ones. To avoid this bias, it should be necessary to calculate selective confidence limits for an arbitrary number of ranks of areas of decreasing size. We have chosen a different approach: to normalize individual ITV values to remove the fraction of ITV inversely related to area using the regression line provided by experimental data. As exemplified in Figs 4 and 5, this procedure, simple and fast by using purpose made Excel sheets, makes all ITV values directly comparable with each other, allows the use of common confidence limits regardless of the size of individual areas and prevents overrating the changes of pathological responses of small size.

### Complex nature of r-CMAP and MEP

Regardless of the label of recording site, any r-CMAP or MEP originates from a non-selective root or transcranial stimulation. As a consequence, it is always the result of a complex “cross-talk” interaction between several muscles simultaneously activated without any functional or myelomeric pattern. For example, VM responses are generated not only by VM, but partly also by nearby muscles, including the large posterior muscles of the thigh, with unpredictable additive or subtractive effects. Also motor responses from PL are likely to be affected from the adjacent and larger TA and from the calf muscles, particularly the lateral gastrocnemius. Likewise, most of the small plantar muscles of the foot may partly contribute to generate FHB responses [33]. These complex functional conditions are likely to play a significant role in area variability.

The topographic pattern of muscle recruitment is quite similar for root and for facilitated transcranial stimulation: in both cases a single massive volley (root stimulation) or multiple descending volleys (TMS), impinge on the same muscle districts without any functional pattern. This makes the ratio between MEP and r-CMAP areas an index of conduction failure methodologically more correct than that between a MEP and a CMAP area recorded from the same site but elicited by a selective, distal stimulation of the corresponding peripheral nerve.

Since, during voluntary facilitation, TMS virtually excites all motor neurons [3], the area of r-CMAP to maximal root stimulation should represent the maximum theoretical area of the MEP recorded from the same muscle district, in absence of desynchronization within the motor pathway (which reduces MEP area) or multiple firing of spinal motor neurons (which increases MEP area). The mean TA a-Ratio nearly reached this maximum theoretical values, possibly indicating a good balance between the two above opposing factors, while the lower a-Ratio values (about half) for VM, VL and PL may reflect either a prevailing effect of desynchronization or a reduced or absent repetitive firing by spinal motor neurons.

### ITV and RIV values

RIV and ICC are two common methods of analysis employed to assess reliability of repeated measurements. Very large ICC values were obtained for both latency indexes, associated with low RIVs, and for area indexes, associated, as expected, with larger RIVs. A low RIV, much more than a large ICC, reflects a good reproducibility of a parameter in repeated determinations, and it is a shared opinion that RIVs lower than -10% to +10% represent measurements of high precision [16]. To date this target has been reached only by the F wave latency, considered the most reproducible measure in nerve conduction studies [15,16]. This mainly occurs because, as already emphasized, the F wave explores a very long conduction distance, as it is the case for r-CMAP and MEP latencies recorded from distal muscles. Except for VL r-CMAP, all RIVs for r-CMAP and MEP latencies are fully within the optimal RIV threshold. RIVs for CMCT were slightly greater, because this value includes the variability of both r-CMAP and MEP latencies.

Area-ratio, the most important neurophysiological index to detect conduction failure, showed RIV values not very far from the optimum range (at least for FHB) and with a clear proximo-distal gradient, being RIVs from proximal muscles (VM and VL) greater than RIVs from distal districts (FHB). This may suggest a more relevant role of cross-talk phenomena in proximal districts as compared to distal ones. Our results are not comparable with other published data because ITV for MEP or CMAP areas has never been quantified. Only ITV of the amplitude of CMAPs to median, ulnar, tibial nerves stimulation were reported [15,16] and the resulting RIVs were much higher than that of latencies.

### Clinical prospects of the procedure

As a diagnostic tool, the technique provides 3 latency (r-CMAP, MEP and CMCT) and 3 area (r-CMAP, MEP and a-Ratio) indexes for each of the 10 (5 per side) recording sites, i.e. 60 neurophysiological motor parameters collected from each subject in a single recording session. Clinical data must be obviously referred to normative values obtained from healthy volunteers.

In this short-term follow-up study, normative ITV values were obtained from a casuistry consisting of normal subjects and clinically stable MS patients equally distributed. As a matter of fact, we could not know in advance if short-term variability were the same in healthy subjects and in clinically stable MS patients, as it was proven by our results.

## Conclusions

Magistris et al. [3] first emphasized the potential value of MEP area to quantify motor conduction failure as the neurophysiological index more directly correlated to motor impairment. The “triple stimulation technique” (TST) was proposed as the only effective mean to eliminate chronodispersion within the cortico-spinal pathway and make the ratio between MEP and peripheral CMAP areas (a-Ratio) a precise *measure* of conduction failure. In lower limbs, TST can be applied in distal muscles, which must be examined one by one [34].

Unlike TST, our approach does not remove chronodispersion, but simply perform a satisfactory “smoothing” of this biological bias, as proved by the faithful intertrial replication of the shape of averaged MEPs. In addition to MEP averaging, this goal was allowed by the use of constant recording sites and by an objective procedure of voluntary facilitation. This methodological approach legitimates our a-Ratio as a reliable *index* of conduction failure.

On the other hand, TST totally removes not only physiological, but also pathological chronodispersion, which reflects a differential conduction slowing within the corticospinal fiber population and results in prolonged and scattered MEPs. The morphological features of these abnormal responses, simultaneously monitored by our technique in several proximal and distal muscle districts of both sides, represent useful neurophysiological data, helping to better define the specific pathophysiological profile of motor impairment in individual patients.

## Supporting Information

S1 Appendix Procedure for ITV normalization. (DOCX)

S2 Appendix Individual values of r-CMAP and MEP latencies and areas. (XLSX)
