# Supplementary material for: Normative Values for Intertrial Variability of Motor Responses to Nerve Root and Transcranial Stimulation: A Condition for Follow-Up Studies in Individual Subjects
Source: PLoS One. 2016 May 16;11(5):e0155268. doi: 10.1371/journal.pone.0155268 (PMC4868303; doi:10.1371/journal.pone.0155268)
Supplement: S1 Appendix — (DOCX) [file pone.0155268.s001.docx]

**Appendix: normalization procedure**

**Calculation of the constant “CV” (coefficient of variability), expressing the inverse correlation between area and ITV.**

Plotting individual ITV values related to the respective area values (mean of the two trials) defines a regression line, which expresses the strength of their inverse correlation (see results). To obtain the constant “*CV*” first we calculated the ratio between the extreme ITV values identified by the regression line (ITV max, ITV min, %) and then we divided the obtained result for the difference between the extreme area values observed in our casuistry (∆*AREA****,*** mVmsec), according to the following formula:

*CV* (%) = 100 x (1- ITVmax/ITVmin)

∆*AREA*

*CV* represents the ITV component inversely related to area for each unit of area (1 mVms). The resulting *CV* values were 0.42, 0.34, 0.66 for r-CMAP, MEP and a-Ratio respectively.

**ITV normalization**

We identified the maximum theoretical area value (*p*= 0.001) for each parameter (r-CMAP, MEP and a-Ratio) as the mean area values from our casuistry + 3SD =
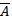
. The resulting values for
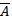
 were 112, 85, 93 mVms for r-CMAP, MEP and a-Ratio respectively. Note that for a-Ratio, “area” is the mean between 4 area values (r-CMAPs and MEPs of the first and second session).

To perform normalization of a given rawITV value *n* *(*raw ITV*n)* of a given Area *n* (A*n*) (mean of the two trials), we multiplied the unitary constant of variability *CV* for ∆A*n* , where ∆A*n =*
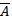
 - A*n* , according to the following formula

Norm ITV*n* = raw ITV*n* x 100 – (*CV* x ∆A*n*)

100

Two examples of normalization are presented, referring to ITVs of a small (a.) and a large (b.) r-CMAP area. (*CV:* 0.42;
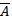
 value: 112).

***a.*** r-CMAP of 8.2 mVms with a raw ITV of 49.7% (maximum theoretical r-CMAP area 112mVmsec; ∆A*n* = 112 - 8.2 = 103.8

n-ITV = 49.7% x 100 - (0.42 x 103.8) = 28.03%

100

***b.*** r-CMAP of 93.5 mVms and a raw ITV of 51.2%; ∆A*n* = 112 – 93.5 = 18.5

norm-ITV = 51.2% x 100 - (0.42 x 18.5) = 47.20%

100

The two examples clearly show that ITV reduction due to normalization is greater for a response of small size (28.03% vs 49.7%) as compared to a response of large size (47.20% vs 51.20%)

The same procedure was applied to normalize MEP and a-Ratio ITVs using the respective
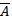
 and *CV* values. The use of a custom made Microsoft Excel ® sheet simplifies and speed up the normalization procedures.
